# Toxicological Characterization of Six Plants of the Beninese Pharmacopoeia Used in the Treatment of Salmonellosis

**DOI:** 10.1155/2019/3530659

**Published:** 2019-07-01

**Authors:** Boris Legba, Victorien Dougnon, Esther Deguenon, Jerrold Agbankpe, Maximin Senou, Alidah Aniambossou, Carène Gbaguidi, Kevin Sintondji, Lamine Baba-Moussa, Jacques Dougnon

**Affiliations:** ^1^Research Unit in Applied Microbiology and Pharmacology of Natural Substances, Research Laboratory in Applied Biology, Polytechnic School of Abomey-Calavi, University of Abomey-Calavi, 01 P.O. Box, 2009 Cotonou, Benin; ^2^Laboratory of Molecular Biology and Typing in Microbiology, Faculty of Science and Technology, University of Abomey-Calavi, 05 P.O. Box, 1604 Cotonou, Benin; ^3^Laboratory of Clinical and Experimental Biology, Faculty of Sciences and Techniques of Dassa, National University of Sciences, Technologies, Engineering and Mathematics, Benin

## Abstract

Recent studies reported interesting ethnopharmacological, antibacterial, and phytochemical data on some medicinal plants used in the traditional treatment of salmonellosis in Benin. Unfortunately, very little data exists on the toxicity of these species. This study aims to evaluate chemical characteristic of six Benin pharmacopoeial plants used in the traditional treatment of salmonellosis in Benin. The acute toxicity of aqueous and ethanolic extracts of* Psidium guajava*,* Vernonia amygdalina*,* Cajanus cajan*,* Phyllanthus amarus*,* Uvaria chamae*, and* Lantana camara* was evaluated according to OECD Guideline 423 at a single dose of 2000 mg/kg body weight on Wistar rats. Histological sections were performed on the liver and kidneys to confirm hematological and biochemical data. The content of aluminum, chromium, cadmium, copper, iron, lead, zinc, arsenic, selenium, and manganese was measured in 10 mg of each extract by the inductively coupled plasma optical emission spectroscopy (ICPOES) method. The results of our study generally show the absence of significant effect of the extracts on the hematological and biochemical parameters of the rats. However, with the exception of the aqueous and ethanolic extracts of* Psidium guajava* root and the ethanolic extract of* Phyllanthus amarus *(P>0.05), all the extracts have a significant effect on the aspartate aminotransferase (ASAT) level, with a variable threshold of significance (0.0001< P ≤ 0.05). No mortalities and no renal histological conditions were recorded in the treated rats. In general, the heavy metal contents of the extracts do not exceed the standards set by the WHO/FDA except for a few extracts. Arsenic was not detected in any extract, while aluminum and chromium were detected at levels above the WHO/FDA standards. On the basis of these data, it appears that the six plants studied do not show any toxicity. In view of the pharmacological and chemical data previously available, these plants are good candidates for the development of improved traditional medicines with antibacterial and particularly anti-*Salmonella* properties.

## 1. Introduction

Humanity faces all sorts of conditions and the handling of health issues is a real social problem, especially in developing countries with limited resources [1]. Recourse to readily available local resources seems to be a real palliative solution from a sustainable development perspective [2]. Despite advances in biology and medicine, the majority of people in developing countries do not have access to adequate healthcare [3]. For this reason, plant resources occupy a large place in the lives of these populations [4].

The African continent has an impressive ethnopharmacological potential consisting of a variety of medicinal plants. According to World Health Organization, more than 80% of African populations use medicine and traditional pharmacopoeia to solve health problems [5]. On more or less 300,000 species of medicinal plants listed on the planet, more than 200,000 develop in the tropical countries of Africa and possess medicinal virtues [5]. In this arsenal, plants used to treat diarrheal diseases, in this case salmonellosis, occupy an important place. Indeed, this disease poses a public health problem in many countries, because it is an important cause of mortality. Medicinal plants are an ideal alternative to chemical drugs that are too expensive to manufacture or buy for developing countries.

For rational use of traditional pharmacopoeia, much research is being done on traditional recipes to obtain improved traditional medicines. However, any biologically active substance is susceptible, depending on the dose, to produce undesirable or even harmful effects. Similarly, the evaluation of the acute general toxicity of the extract and the determination of biocontaminants such as heavy metals are necessary to determine the tolerance limits of the plant.

In Benin, ethnopharmacological studies realised by Dougnon et al. [6, 7] identified 114 species of medicinal plants used by herbalists, traditherapists, and breeders in the management of salmonellosis in southern Benin. The most cited of these species have been valued for their antibacterial properties, data on their composition of bioactive substances, and their larval toxicity [8] which allowed selecting six most active plants. Unfortunately, the acute toxicity and the content of heavy metals of these plants are almost nonexistent or insufficient. It is this gap that this research work aims to fill. The aim is to evaluate the acute toxicity and the heavy metal content of the aqueous and ethanolic extracts of six (6) Benin pharmacopoeia plants used in the treatment of salmonellosis. These data will be used to attest to the safety of plant extracts and will strengthen data on their biological activities and chemical composition.

## 2. Materials and Methods

Several research institutions have served as a framework for the study:

(i) The Laboratory of Biology and Molecular Typing in Microbiology, Faculty of Sciences and Technology (University of Abomey-Calavi, Benin), served as a framework for extraction.

(ii) The Research Unit in Applied Microbiology and Pharmacology of Natural Substances, Polytechnic school of Abomey-Calavi (University of Abomey-Calavi, Benin), served as a framework for acute toxicity testing.

(iii) The Chemistry Laboratory of the Department of System Innovation of the Graduate School of Engineering of the University of Tokyo served as a framework for the determination of heavy metals.

### 2.1. Materials

#### 2.1.1. Plant Material

The plant material consists of the organs of six (6) medicinal plants of the Benin pharmacopoeia used in the traditional treatment of salmonellosis in Benin (Table 1). After their identification at the National Herbarium (University of Abomey-Calavi) by Professor Hounnankpon Yedomonhan, leaf samples were cleaned and dried at laboratory temperature to better preserve the heat-sensitive molecules. These dried plants were then milled using a SM 2000/1430/UPM/SMF type Retsch Mill.

#### 2.1.2. Animal Material

The animal material consists of albino rats Wistar weighing between 200 and 250g. They were acquired from the animal facility of the Institute of Applied Biomedical Sciences in Benin. Upon receipt, the rats were randomly divided into lots of 3 in standard cages for an acclimation period of 2 weeks prior to use. During this period, the animals had free access to food and water. They were kept at the pet shop at a constant temperature of 22 ± 2°C and subjected to a 12/12h light/dark cycle.

### 2.2. Methods

#### 2.2.1. Extracts Preparation

The total aqueous and ethanolic extracts were prepared. Fifty grams of powder was macerated in 500 ml of distilled water or ethanol on a Stuart Bioblock Scientific Fisher shaker for 72 hours at room temperature. The homogenate obtained was filtered three times on hydrophilic cotton followed by filtration on Whatman No. 1 paper. This filtrate was then dried at 45°C in a Pasteur oven.

#### 2.2.2. Acute Toxicity Test and Treatment of Rats

Rats were divided into lots according to their weight. The extracts were administered orally according to the method described in OECD Guideline 423 (Organization for Economic Cooperation and Development). Since plant species are commonly used by the public and no toxic effects were identified, a limit toxicity test of a single dose of 2000 mg/kg body weight was used. Twelve hours before the toxicity tests were carried out, the animals were deprived of food and water. After the weighing of the rats, three lots of three rats were constituted and distributed as follows:

 
*Lot 1*: control lot receives only distilled water.
 
*Lot 2*: aqueous extract of the leaves of* Psidium guajava*
 
*Lot 3*: ethanolic extract of the leaves of* Psidium guajava*
 
*Lot 4*: aqueous extract of the roots of* Psidium guajava*
 
*Lot 5*: ethanolic extract of the roots of* Psidium guajava *
 
*Lot 6*: aqueous extract of the leaves of* Vernonia amygdalina*
 
*Lot 7*: ethanolic extract of leaves of* Vernonia amygdalina*
 
*Lot 8*: aqueous extract of the leaves of* Cajanus cajan *
 
*Lot 9*: ethanolic extract of the leaves of* Cajanus cajan *
 
*Lot 10*: aqueous extract of the leaves of* Phyllanthus amarus*
 
*Lot 11*: ethanol extract of the leaves of* Phyllanthus amarus*
 
*Lot 12*: aqueous extract of the leaves of* Uvaria chamae*
 
*Lot 13*: ethanol extract of the leaves of* Uvaria chamae*
 
*Lot 14*: aqueous extract of the roots of* Uvaria chamae*
 
*Lot 15*: ethanol extract of the roots of* Uvaria chamae*
 
*Lot 16*: aqueous extract of the leaves of* Lantana camara*
 
*Lot 17*: ethanolic extract of* Lantana camara* leaves

#### 2.2.3. Post treatment

After treatment, the rats were monitored and observed individually every thirty minutes during the day and then daily for 14 days. An information collection card was drawn up for each rat in order to collect any signs of toxicity (changes in the skin, hair, eyes, appearance of oedema, as well as the respiratory system, death). The following examinations were conducted.

*Hematological Examinations*. The hematological examinations were carried out using a SYSMEX KX 21N. These included red blood cell count, white blood cell count, and determination of the hemoglobin (Hb), hematocrit (HTE), mean corpuscular volume (MCV), the mean corpuscular hemoglobin (MCH), and mean corpuscular hemoglobin concentration (MCHC).

*Biochemical Examinations*. These were urea, creatinine, and transaminases.

*Histological Examinations*. Histological sections were made at the histopathology laboratory of ISBA and the pathomorphological study consists of the staining with hematoxylin of thin sections of 5 *μ*m thickness and observation under an optical microscope. Histological analysis of the liver and kidney was performed according to the basic techniques described by Smith and Bruton (1977).

The organs were previously fixed in Bouin's liquid and in 10% buffered formalin.

#### 2.2.4. Removal of Organs

During this step, the livers and kidneys were removed and fixed with Bouin (formalin + picric acid + acetic acid) and 10% buffered formalin. The resulting slices were placed in carefully labeled histological cassettes (3.0 x 2.5 x 0.4 cm).

#### 2.2.5. Traffic

This stage has three substeps: dehydration, lightening, and impregnation. The dehydration consisted of ridding the tissue of all traces of water (the Bouin liquid being aqueous and the water immiscible in impregnation/inclusion medium that is paraffin). To achieve this, the biopsies were kept in ethyl alcohol baths (organic dehydration solvent) of increasing concentrations: at 70° for one hour, at 80° for one hour, at 95° for one hour, and this three times, continued at 100° for 45 minutes twice in a row. The lightening consisted of replacing the dehydrating solvent with a so-called “transition” solvent miscible with paraffin: toluene.

To do this, the tissues stayed in two xylene baths, each for one hour. The impregnation consisted of replacing the transition solvent with paraffin to make them softer and easier to cut. To do this, the tissues stayed in two successive baths of melted paraffin, each for one hour.

#### 2.2.6. Inclusion

Inclusion provided an external support for the tissues, which allows a good realization of microtome sections. After wax impregnation, the tissues were removed from the cassettes, introduced into molds filled with melted paraffin, and solidified on a cold surface after proper orientation of the tissue in the block.

#### 2.2.7. Histological Sections

The sections of the different blocks were made with a microtome at a thickness of 3 microns. Once cut, the sections forming a paraffin ribbon are deflowered in a bath of distilled water at 40°C and then each recovered on a microscopic slide previously degreased with alcohol. The different blades thus formed will be put in an oven at 55°C for at least 24 hours.

#### 2.2.8. Staining

This step allowed us to highlight the different tissue elements and differentiate them; this makes the study of their structure, their morphology, and their pathological modifications possible. To do this, we performed hematoxylin-eosin staining. It is routine staining after which the nuclei, stained by hematoxylin, appear dark blue and the cytoplasm, stained by eosin pink.

#### 2.2.9. Observation and Photography

The slides obtained at the end of the staining were observed under an Olympus optical microscope and photographs were taken using a camera adapted to the microscope. The analysis of the slides was done so that only the most representative photographs were selected.

### 2.3. Dosage of Heavy Metals

Induced inductively coupled plasma optical emission spectroscopy (ICPOES) was used to search for aluminum, chromium, cadmium, copper, iron, lead, zinc, arsenic, and selenium.

#### 2.3.1. Preparation of Samples

Acid digestion is then with 10 mL of HNO3-HCl-H2O2 (8: 1: 1, v/v/v). The tubes were placed on a heating block with the temperature set to increase to 120°C for about 3 hours or until the solutions were completely digested. An acid digest of each plant was prepared by oxidizing 10 mg of extract. 1mL of the acid digests of each sample was further diluted to 20 ml due to the corrosive nature of the acid used. Each extract was filtered 3 times to remove impurities and used for reading on ICP-OES.

#### 2.3.2. Analysis by ICPOES Technique

The methodology and an adaptation of that were used by Sung [9]. The heavy metal content of the extract T is determined according to the formula T = C ∗V/m (mg/g) with the error E = SD ∗ V/m. With T (Heavy metal content), C (concentration of heavy metal in the extract), m (mass of extract), V (volume of acid digest solution).

### 2.4. Statistical Analysis

GraphPad Prism version 7.0 software was used for statistical testing. The acute toxicity assessment consisted of averages and standard deviations. Using the nonparametric Mann-Whitney test, the means of the Control Lot were compared with those of the other lots. A significance level of 5% was applied for the tests performed. The following scale was used to assess the level of significance (Table 2).

## 3. Results and Discussion

### 3.1. Results

#### 3.1.1. Acute Toxicity of Extracts

Throughout the experiment the animals remained alive. 24 hours after the administration of extracts, a state of temporary agitation was observed, followed by calmness. The hematological parameters were determined on days 0, 7, and 14. The results of the Test t-student concerning the evolution of the number of white blood cells and red blood cells are presented in Table 3. No significant difference was observed between the values of the control batch and those of the extracts tested. The results of the Test t-student on changes in hemoglobin (Hb), hematocrit (HTE), mean median volume (MVV), mean hemoglobin content (MHC), and mean corpuscular hemoglobin concentration (MCHC) are presented in Table 4.

**Figure 1:**
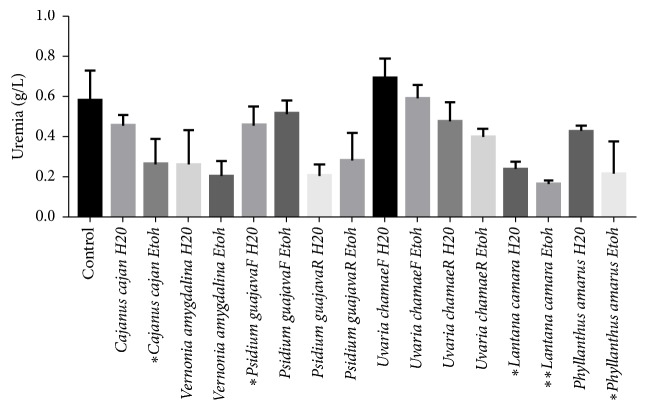
Effects of extracts on uremia.

**Figure 2:**
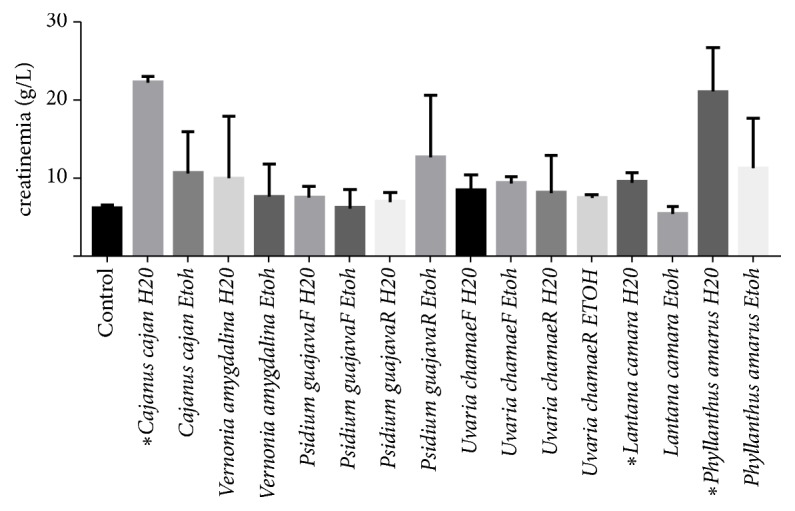
Effects of extracts on creatinine.

**Figure 3:**
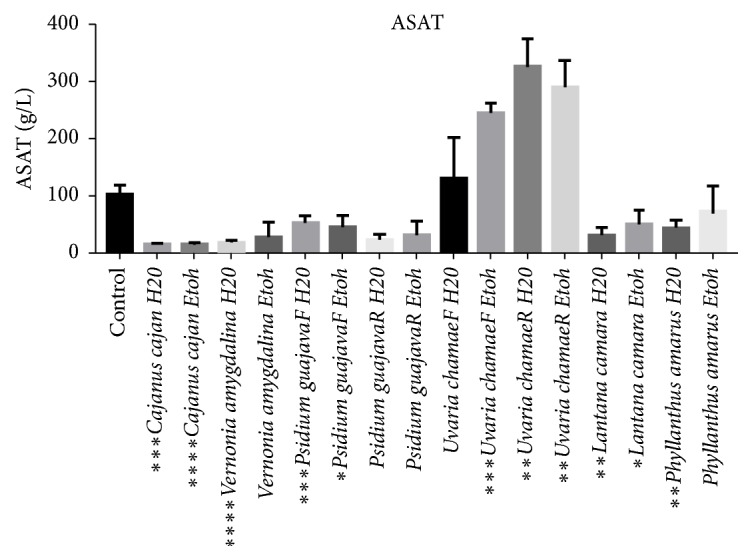
Effects of extracts on aspartate aminotransferase.

**Figure 4:**
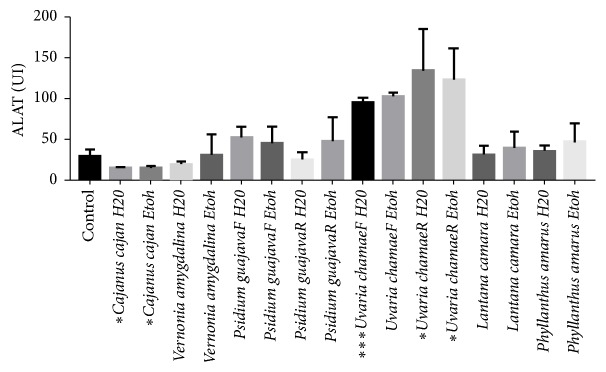
Effects of extracts on alanine aminotransferase.

Biochemical parameters were also evaluated. These are uremia (Figure 1), creatinemia (Figure 2), ASAT (Figure 3), and ALAT (Figure 4). The aqueous extracts of* Cajanus cajan*, the aqueous and ethanolic extracts of* Vernonia amygdalina*, the ethanolic extracts of* Psidium guajava* leaves, the aqueous and ethanolic extracts of* Psidium guajava* roots, the aqueous extracts of the leaves and roots of* Uvaria chamae*, and the aqueous extracts* Phyllanthus amarus* have no significant effect on the uremia of treated rats (Figure 1).

With the exception of aqueous extracts of* Cajanus cajan, Lantana camara, and Phyllanthus amarus*, which have a significant effect on creatinemia, none of the other extracts of the plants studied had a significant effect (P>0.05) (Figure 2).

With the exception of the aqueous and ethanolic extracts of* Psidium guajava* root and the ethanolic extract of* Phyllanthus amarus*, all extracts have a significant effect on the ASAT level, with a variable significance threshold (Figure 3).

With the exception of aqueous and ethanolic extracts of* Cajanus cajan*, aqueous extracts of* Uvaria chamae* leaves, and aqueous and ethanolic extracts of* Uvaria chamae* roots, all extracts have a nonsignificant effect on ALAT levels.

**Figure 5:**
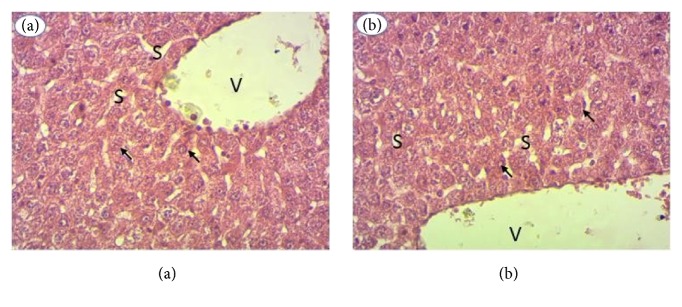
Liver histology, 400x magnification. (i) V: centrilobular vein. (ii) S: sinusoids.

**Figure 6:**
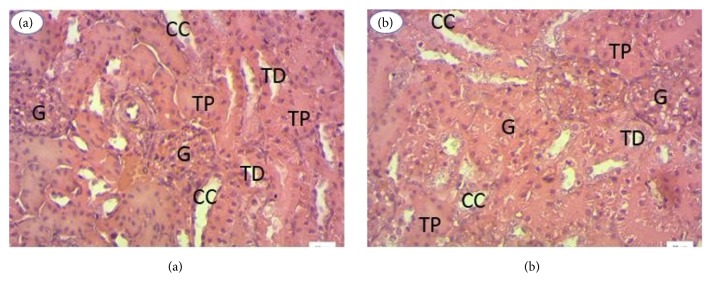
Renal histology, 400x magnification. (i) CC: collecting ducts. (ii) G: the glomeruli. (iii) TP: the proximal tubes. (iv) TD: the distal tubes.

From the tissues of the kidneys and the liver, histological sections were made to confirm the hematological and biochemical data. For all extracts, histological sections of the organs of the treated rats showed no structural abnormality compared to the controls.  Figure 5 shows liver histology of* Cajanus cajan*-treated rats (Figure 5(b)) and control rats (Figure 5(a)). The liver parenchyma of treated rats (Figure 5(b)) has a typical appearance as observed in control rats (Figure 5(a)). The hepatocytes (arrows) have a normal appearance and are arranged in cords separated by the sinusoids (S). The sinusoids flow into the centrilobular vein (V).  Figure 6 shows the liver histology of rats treated with* Cajanus cajan* (Figure 6(b)) and control rats (Figure 6(a)). The renal parenchyma of treated rats (Figure 6(b)) has the typical architecture observed in control rats (Figure 6(a)). The glomeruli (G), the proximal tubes (TP), the distal tubes (TD), and the collecting ducts (CC) are clearly identifiable. The extract therefore has no effect on renal structures. The same result was observed for all extracts.

#### 3.1.2. Heavy Metals Content of the Extracts

Table 5 shows the heavy metal content of the extracts (mg/mg) and the standard tolerated values (mg/mg) by the WHO and the FDA (Food and Drug Administration) [10]. For each chemical element, the best wavelength was chosen. This is Al396.153; Cd228.802; Cr205.56; Cu 327,393; Fe238.204; Pb_2_20.353; Zn213.857; Se196.026; As188.979; Mn257.610.

For the measured contents, the negative values are indicated as “under detection level (UDL)”. In fact, the measuring instrument cannot measure a concentration lower than 0.001 mg/L. In general, the extracts have a variable content of heavy metals. The aluminum and chromium content detected in all extracts is above the average value tolerated by WHO and FDA. For cadmium (Cd), only* P. guajava* root extracts,* Uvaria chamae* leaf extracts, and ethanolic leaf extracts of* Phyllanthus amarus* have a level above the tolerated value. With respect to copper, except for aqueous extracts of leaves and root extracts of* Psidium guajava*, heavy metal was not detected in any extract, which meets WHO standards. With regard to iron (Fe), six of the extracts have a content above the tolerated value. These are leaf extracts of* Vernonia amygdalina*, ethanolic extracts of* Psidium guajava* leaves, aqueous extracts of* U. chamae* root, and aqueous extracts of leaves of* Phyllanthus amarus*. For lead, the aqueous extracts of* Lantana camara* leaves, the ethanolic extracts of* P. guajava* leaves, and the aqueous extracts of* Vernonia amygdalina* leaves have a content above the tolerated limit. As regards zinc, except the ethanolic extracts of* U. chamae* roots for which the metal has not been detected, all the extracts have a content above the tolerated limit. For selenium, except aqueous extracts of* Phyllanthus amarus* leaves,* Lantana camara*, and ethanolic extracts of* Cajanus cajan* leaves, the metal was not detected in any extract. Arsenic was not detected in any of the extracts, which is in line with WHO standards. Only leaf extracts of* Cajanus cajan, Vernonia amygdalina, *and* Psidium guajava*, ethanolic root extracts of* Psidium guajava *and* Uvaria chamae*, and ethanolic extracts of* Phyllanthus amarus* leaves comply with WHO/FDA standards for manganese.

### 3.2. Discussion

This study aimed at the toxicological characterization of the extracts of six medicinal plants used in the traditional treatment of salmonellosis in Benin.

#### 3.2.1. Acute Toxicity of Extracts

The dose was single (2000 mg/kg body weight). Aqueous and ethanolic extracts of* Cajanus Cajan* had no significant effect on hematological and biochemical parameters. This result was supported by histological tests, which showed no renal and hepatic histological abnormalities in the treated rats compared to the control. These observations are similar to those made by Tang et al. [11], which showed a lack of biochemical, hematological, and histological modification following the ingestion of* Cajanus cajan* extracts at a dose of 15.0 g/kg in Kunming mice. Study of Kevin et al. [12] has resulted in the same deductions. In their study, aqueous and hydroethanol extracts of* Cajanus cajan* were not toxic at maximum tolerated doses (MTD) of 2500 mg/kg and 400 mg/kg body weight, respectively. Excluding mean corpuscular volume (MCV), mean corpuscular hemoglobin concentration (MCHC), and ALAT,* Vernonia amygdalina* extracts had no significant effect on the biochemical and hematological parameters of treated rats compared to control. No lesions were also observed in the liver and kidneys histologically. At the dose tested, the extracts therefore do not exhibit toxicity. These data confirm the observations made by Zakaria [13], which show that, even up to the dose of 5000 mg/kg, the aqueous extracts of the leaves of* Vernonia amygdalina* do not cause any mortality in the Wistar rats and no significant modification in biochemical and hematological parameters. Ekpo [14] also reached the same conclusions from biochemical data.

The aqueous and ethanolic extracts of* the Psidium guajava* organs did not induce any mortality in the treated rats. Hematologically, no significant effect was observed except in the mean corpuscular volume (MCV) where the ethanolic root extract induced a significant increase in volume. On the biochemical level, no significant influence of the extracts of this plant on the biochemical parameters was noted, but the ethanolic extracts of roots led to a significant increase of the uremia and the rate of ASAT. In addition, histological data show that renal and fetal tissues have not been affected. The nontoxic nature of the aqueous leaf extract of* Psidium guajava* has already been reported for a dose of 10-50 mg/100 g in Wistar rats [15]. Regarding the significant effect of ethanolic root extract on biochemical parameters, some literature data support our results. It has been reported that ethanolic extracts of* Psidium guajava* root significantly increase serum enzymes such as ALAT and ASAT in a subchronic study for a dose of 150-1200 mg/kg [16].

In general, the aqueous and ethanolic leaf and root extracts of* Uvaria chamae* generally had no significant effect on the hematological parameters of the treated rats except for the leaf extracts which induced a significant increase in the volume of the mean corpuscular volume (MCV). At the biochemical level, no significant effect is observed at the level of uremia and creatinemia but a sharp increase of the rate of ASAT and ALAT is noted in comparison with the control. This observation reinforces the one already made by Olumese [17] who showed that at a dose of 5000 mg/kg of body weight the aqueous extracts of roots of* Uvaria chamae* significantly increased the levels of ASAT, and, on the other hand, at 2000 mg/kg of body weight the ALAT level was significantly higher in the treated rats than in the controls. Another more recent study conducted in 2018 by the same authors reports similar observations but this time with ethanolic extracts of* Uvaria chamae* root. For doses between 200 and 5000 mg/kg body weight, a significant increase in ASAT was observed after administration of ethanolic extracts of* Uvaria chamae* roots. It should be recalled that ALAT and ASAT cellular enzymes are present at low concentration in serum under normal conditions. Their increase may have several origins including increased enzyme synthesis or liver failure [18]. Serum ASAT and elevated ALAT levels are considered sensitive markers of possible tissue damage, particularly liver injury [19]. However, in the present study, no histological abnormalities were noted in the liver. It is therefore not possible to assert a possible toxicity due to* Uvaria chamae* root extracts, especially since no mortality of the treated rats is recorded. However, it is necessary to suggest, pending further work, that the extract be used for doses below 2000 mg/kg body weight.

*Phyllanthus amarus* extracts had no particularly significant effect on the hematological parameters of the treated rats, except for the aqueous extract which significantly increased mean corpuscular volume (MCV) and mean corpuscular hemoglobin (MCH). Biochemically, the same extract significantly decreased uremia and ASAT levels in the treated rats compared to the control. These observations converge with those made by Pramyothin [20]. In this study, treatment of rats with aqueous extracts of* Phyllanthus amarus *(75 mg/(kg day), po) for 7 days after 21 days of ethanol treatment (4 g/(kg day), po) brings back levels of ASAT and ALAT to normal, suggesting hepatoprotective properties. Even at 5g/kg body weight, extracts of* Phyllanthus amarus* remain nontoxic as demonstrated by Lawson-Evi et al. [21].

Extracts of* Lantana camara* had significant effects on mean corpuscular volume (MCV) and mean corpuscular hemoglobin concentration (MCHC), serum creatinine, uremia, and ASAT level. These observations without additional data are not sufficient to attribute to the extracts of* Lantana camara*, a toxic character, especially as no mortality was recorded in the treated rats and histological sections revealed no abnormality. Pour et al. [22] also reported significant effect on biochemical parameters, but it is ALAT that has been evaluated. In the book “Toxic Plants” from the Klorane Institute,* Lantana camara* has been listed as a poisonous plant. It is reported that “human intoxications are rare, but the ingestion of drupes causes digestive, respiratory, or neurological disorders, which can go to death in severe cases. Contact with the plant can cause allergic and itchy skin diseases. According to the same source, this toxicity is due to pentacyclic triterpenoids called lantadenes [23]. This toxicity only concerns fruits. The recommendations must be made in this direction for the people.

#### 3.2.2. Heavy Metal Content of the Extracts

The heavy metal content was evaluated to supplement the acute toxicity data established by this study and the initially available data on the toxicity of the extracts (larval toxicity and subchronic toxicity). The aim is to optimize the toxicological characterization in order to provide complete data for optimal recovery, without however overshadowing the fact that the accumulation of heavy metals does not only have harmful consequences.

The extracts of the six medicinal plants studied have a variable composition of heavy metals. Leaf extracts of* Psidium guajava* have lead, chromium, and aluminum levels above the limit value. Similar observations have already been made on this same species harvested in some mining areas of Katanga province in the Democratic Republic of Congo, with however much higher rates (probably due to the fact that the study site is a mining area). It should be recalled that, in this study, the rate of this metal in the species varied from one site to another, reflecting the environmental factor highly involved in the presence of heavy metals [24]. It also results that it is useless to compare this rate with that of other studies as long as the environment of origin is not the same. The results obtained with the extracts of* Lantana camara* are very divergent from one extract to another with regard to lead. The aqueous extract has a lead content above the limit value, while the ethanolic extract has a value below the tolerated limit value. The low accumulation of lead by* Lantana camara* has already been reported by Khankhane and Varshney [25]. The divergence in the extracts can be attributed to an influence of the solvents or to the sensitivity of the detection method. The same observations can be superimposed on the data of the aqueous and ethanolic extracts of* Phyllanthus amarus*, with regard to iron and cadmium. However, studies in Ghana on crude powders show relatively high levels of iron and, conversely, values below the WHO/FAO limit for cadmium [26].

The presence and concentration of heavy metals in medicinal plants can be related to several factors including the source (soil), the physicochemical properties of the soil that affect the concentration of heavy metals [27], pH, temperature, oxidation-reduction potential, cation exchange capacity and organic matter [28], and interactions between roots and soil microbes [29].

The presence of heavy metals in medicinal plants, above a certain threshold, announces probable toxic effects. These toxic effects are due to their hindrance in normal metabolic processes. Arsenic and chrome are frequently involved in morbidity and death in South Africa [30]. A study of the forensic record between 1991 and 1995 in Johannesburg identified 206 cases in which a traditional remedy had either been declared a cause of death or found present in a case of poisoning with an unidentified substance [31]. Heavy metals are responsible for 10% of these poisonings. A study of patients treated with traditional remedies showed that, on 12 concoctions studied, copper (Cu) concentrations were extremely high in four concoctions. 34% of patients had elevated zinc (Zn) concentrations and one patient had a Zn concentration 10 times higher than normal. After a week of vomiting with hepatomegaly and dehydration, the patient died of liver failure [32]. In another study, it was determined that a seven-month-old infant had been hospitalized after taking a traditional drug, resulting in a severe case of multimetal intoxication [30].

The presence of heavy metals in plant extracts does not only have harmful dimensions. For example, it can influence the production and structure of secondary metabolites of plants. It has been shown that* Phyllanthus amarus'* therapeutically active phyllanthine and hypophyllanthin compounds are enhanced at certain levels of Cd stress [33]. It is in the same sense that it has been suggested that some medicinal plants are grown in polluted soils to achieve a higher secondary metabolite yield [34].

Some authors believe that the term “contamination” used for the presence of heavy metals in medicinal plants is misleading [35]. This assertion is supported by the fact that in some pharmacopoeias heavy metals are used in the management of many health problems. In Chinese medicine, mercury is part of certain preparations referred to as “cinnabaris” (mercury sulphide), “calomel” (mercury chloride), or “hydrargyri oxydum rubrum” (mercury oxide) [35]. Similarly, in Indian traditional medicine, bhasma (calcified powder/ash) is a herbal-mineral or herbal metal formulation [33].

## 4. Limitations

Although this study provides interesting data on the toxicity of the plants studied, the median lethal dose was not determined and the toxicity was not evaluated over a long period of time.

## 5. Conclusions

The extracts of the six medicinal plants studied did not induce any mortality or any histological renal and fetal disorder in the rats treated in the acute toxicity test, although in some places there was a significant effect on the biochemical and hematological parameters. These extracts cannot be attributed to toxicity at a dose of 2000 mg/kg. The contents of heavy metals vary from one extract to another. Some grades are below tolerable levels, while others are well above them. It is important to evaluate the health risks associated with the presence of each heavy metal in order to make objective recommendations. Otherwise, it is very important to determine median lethal dose and complete these data by subacute toxicity.

## Figures and Tables

**Table 1:** Composition of plant material.

N°	Species of plants	Organs used	Collection site	Specimen voucher number
1	*Psidium guajava*	Roots and leaves	Abomey-Calavi	AA6694/HNB
2	*Vernonia amygdalina*	Leaves	Abomey-Calavi	AA6662/HNB
3	*Cajanus cajan*	Leaves	Abomey-Calavi	AA6698/HNB
4	*Phyllanthus amarus*	Leaves	Adjarra	AA6686/HNB
5	*Uvaria chamae*	Roots and leaves	Adjarra	AA6687/HNB
6	*Lantana camara*	Leaves	Adjarra	AA6688/HNB

**Table 2:** Assessment of the significance.

Symbol	P value	Deduction
Ns	P> 0.05	Not significant
*∗*	0.01< P ≤ 0.05	significant
*∗∗*	0.001< P ≤ 0.01	very significant
*∗∗∗*	0.0001< P ≤ 0.001	highly significant
*∗∗∗∗*	P ≤ 0.0001	Very Highly significant

**Table 3:** Changes in the levels of white cells and red blood cells in the treated and control rats.

	Red Blood cell	White Blood cell
Control	11.03 ± 2.395	7.39 ± 0.6701
*Cajanus cajan h20*	8.867 ± 1.445	6 ± 0.1155
*Cajanus cajan Etoh*	5.467 ±0.333	6.197 ± 0.8261
*Vernonia amygdalina H20*	11.67 ± 4.278	5.32 ± 0.7407
*Vernonia amygdalina Etoh*	7.1 ± 2.163	6.27 ± 0.7087
*Psidium guajavaf H20*	10.97 ± 2.14	5.017 ± 1.197
*Psidium guajavaf Etoh*	6.867 ± 2.354	5.85 ± 1.351
*Psidium guajavaR H20*	9 ± 0.5774	8.133 ± 0.4096
*Psidium guajavaR Etoh*	10.73 ± 1.322	7.883 ± 0.3768
*Uvaria chamaef H20*	7.533 ± 0.4842	6.367 ± 0.5608
*Uvaria chamaef Etoh*	7.2 ± 0.9504	5.837 ± 0.09135
*Uvaria chamaeR H20*	6.47 ± 0.09074	6.403 ± 0.1184
*Uvaria chamaeR Etoh*	6.9 ± 0.7506	5.54 ± 1.08
*Lantana camara H20*	9.3 ± 0.4933	6.9 ± 0.2646
*Lantana camara Etoh*	8.7 ± 0.3512	5.46 ± 0.8746
*Phyllanthus amarus H20*	10.37 ± 0.5897	8.127 ± 0.611
*Phyllanthus amarus Etoh*	8.9 ± 0.8505	6.993 ± 0.3284

**Table 4:** Evolution of the hemoglobin (Hb), hematocrit (HTE) of mean corpuscular volume (MCV), the mean corpuscular hemoglobin (MCH), and mean corpuscular hemoglobin concentration (MCHC) of treated rats and controls.

	Hb	HTE	MCV	MCH	MCHC
Control	13.57 ± 0.7667	45.5 ± 2.627	60.9 ± 2.954	18.77 ± 0.4667	30.87 ± 1.213
*Cajanus cajan h20*	14.5 ± 0.4	42.67 ± 1.202	75.67 ± 1.764*∗*	23.67 ± 0.8819*∗∗*	32.33 ± 1.453
*Cajanus cajan Etoh*	14.57 ± 0.7265	48 ± 1.528	76.67 ± 2.404*∗*	23 ± 0.5774*∗∗*	33 ± 1.528
*Vernonia amygdalina H20*	13.2 ± 0.7024	41.33 ± 1.856	78.67 ± 0.6667*∗∗*	23 ± 0.5774*∗∗*	31 ± 0.5774
*Vernonia amygdalina Etoh*	14.25 ± 0.3994	44 ± 0.5774	77.67 ± 4.807*∗*	25.67 ± 1.202*∗∗*	30.67 ± 1.202
*Psidium guajava f H20*	12.87 ± 0.8192	31.33 ± 5.728	67 ± 4.726	18.43 ± 2.894	32.37 ± 2.421
*Psidium guajava f Etoh*	12.07 ± 1.901	38.77 ± 8.836	66.33 ± 4.429	21.53 ± 2.004	32.6 ± 3.372
*Psidium guajavaR H20*	15.13 ± 0.1202	46.67 ± 4.177	43.33 ± 17.34	27.67 ± 3.18	33.67 ± 0.3333
*Psidium guajavaR Etoh*	15.87 ± 0.4055	51.33 ± 2.028	79.67 ± 0.8819*∗∗*	21 ± 3.606	32 ± 1.155
*Uvaria chamae f H20*	10.77 ± 0.8353	39 ± 2.646	78.33 ± 3.844*∗*	27.97 ± 2.074*∗*	33.4 ± 0.7024
*Uvaria chamae f Etoh*	12.53 ± 0.2728	40.33 ± 2.404	69.67 ± 0.8819*∗*	21 ± 0.7095	30.9 ± 0.4041
*Uvaria chamaeR H20*	13.07 ± 0.8743	39.2 ± 1.513	60.37 ± 5.75	21 ± 0.5686*∗*	36.33 ± 2.362
*Uvaria chamae R Etoh*	11.13 ± 1.923	38.77 ± 0.7667	59 ± 1.155	23.33 ± 0.8819*∗*	36.2 ± 1.361*∗*
*Lantana camara H20*	14.7 ± 0.2082	43.67 ± 0.8819	72.33 ± 6.173	26.67 ± 0.8819*∗∗*	35.33 ± 1.453
*Lantana camara Etoh*	12.73 ± 1.073	36.33 ± 2.404	84 ± 3.055*∗∗*	26.67 ± 0.8819*∗∗*	34.67 ± 1.856
*Phyllanthus amarus H20*	15.9 ± 0.4583	46.33 ± 1.856	80 ± 0*∗∗*	26 ± 1.155*∗∗*	31.67 ± 0.8819
*Phyllanthus amarus Etoh*	14.83 ± 0.6766	40.67 ± 2.186	61.67 ± 4.41	22.67 ± 1.333	33.67 ± 0.8819

**Table 5:** Heavy metal content of the extracts (mg/mg) and tolerated standard values (mg/mg) by the WHO and the FDA (Food and Drug Administration) [9].

	Al	Cd	Cr	Cu	Fe	Pb	Zn	Se	As	Mn
*C.cajan h20*	1.696±0.229	UDL(<0.001)	0.405±0.538	UDL(<0.001)	UDL(<0.001)	UDL(<0.001)	1.340±0.332	UDL(<0.001)	UDL(<0.001)	UDL(<0.001)
*C.cajan Etoh*	1.239±0.338	UDL(<0.001)	0.704±0.293	UDL(<0.001)	UDL(<0.001)	UDL(<0.001)	1.634±0.077	0.252±0.035	UDL(<0.001)	UDL(<0.001)
*V.amygdalina H20*	2.281±0.112	UDL(<0.001)	2.304±0.426	UDL(<0.001)	6.200±0.381	7.625±2;408	5.333±0.160	UDL(<0.001)	UDL(<0.001)	UDL(<0.001)
*V.amygdalina Etoh*	7.924±0.205	UDL(<0.001)	0.517±0.442	UDL(<0.001)	0.052±0.405	UDL(<0.001)	1.349±0.208	UDL(<0.001)	UDL(<0.001)	UDL(<0.001)
*P. guajava f H20*	1.656±0.195	UDL(<0.001)	0.597±0.274	0.299±0.191	UDL(<0.001)	UDL(<0.001)	2.460±0.022	UDL(<0.001)	UDL(<0.001)	UDL (<0.001
*P. guajava f Etoh*	1.072±0.332	UDL(<0.001)	0.796±0.469	UDL(<0.001)	0.022±0.479	0.783±3.152	1.332±0.061	UDL(<0.001)	UDL(<0.001)	UDL(<0.001)
*P. guajava R H20*	1.390±0.031	0.260±0.044	0.968±0.636	3.872±0.250	UDL(<0.001)	UDL(<0.001)	4.506±0.014	UDL(<0.001)	UDL(<0.001)	0.064±0.037
*P. guajava R Etoh*	1.470±0.369	0.070±0.137	0.596±0.453	0.909±0.020	UDL(<0.001)	UDL(<0.001)	3.088±0.078	UDL(<0.001)	UDL(<0.001)	UDL(<0.001)
*U. chamae f H20*	1.709±0.273	0.039±0.084	0.274±0.107	UDL(<0.001)	UDL (<0.001)	UDL(<0.001)	3.197±0.072	UDL(<0.001)	UDL(<0.001)	1.795±0.042
*U. chamae f Etoh*	2.108±0.223	0.009±0.040	0.721±0.508	UDL(<0.001)	UDL (<0.001)	UDL(<0.001)	1.826±0.099	UDL(<0.001)	UDL(<0.001)	0.039±0.011
*U. chamae R H20*	1.468±0.298	UDL(<0.001)	0.541±0.146	UDL(<0.001)	0.446±0.245	UDL(<0.001)	2.112±0.077	UDL(<0.001)	UDL(<0.001)	0.190±0.060
*U. chamae R Etoh*	0.397±0.574	UDL(<0.001)	0.823±0.910	UDL(<0.001)	UDL (<0.001)	UDL(<0.001)	UDL(<0.001)	UDL(<0.001)	UDL(<0.001)	UDL(<0.001)
*L. camara H20*	1.507±0.357	UDL(<0.001)	4.655±0.212	UDL(<0.001)	0.020±0.273	1.466±5.530	4.360±0.156	0.065±0.303	UDL(<0.001)	0.412±0.010
*L. camara Etoh*	1.175±0.325	UDL(<0.001)	0.251±0.611	UDL(<0.001)	UDL (<0.001)	UDL(<0.001)	2.240±0.194	UDL(<0.001)	UDL(<0.001)	UDL(<0.001)
*P. amarus H20*	1.422±0.075	UDL(<0.001)	0.615±0.817	UDL(<0.001)	UDL (<0.001)	UDL(<0.001)	2.671±0.029	0.193±0.656	UDL(<0.001)	0.019±0.028
*P. amarus Etoh*	1.446±0.119	0.013±0.015	0.891±0.390	UDL(<0.001)	0.0641±0.906	UDL(<0.001)	2.520±0.048	UDL(<0.001)	UDL(<0.001)	0.167±0.044
OMS/FDA	UDL	0.003	UDL	UDL	UDL	0.01	UDL	UDL	0.01	UDL

## Data Availability

The data used to support the findings of this study are included within the article.

## References

[1] Mangambu M. (2013). *Taxonomie, biogéographie et écologie des Ptéridophytes de l’écosystème forestier des montagnes du Parc National de Kahuzi-Biega à l’Est de la R.D. Congo [Thèse de doctorat]*.

[2] Mangambu M., Van Diggelen R., Mwanga Mwanga J.-C. (2012). Etude ethnoptéridologique, évaluation des risques d’extinction et stratégies de conservation aux alentours du Parc National de Kahuzi Biega en R.D. Congo. *Geo-Eco-Trop*.

[3] Singh S., Singh R. (2018). Ethnomedicinal use of pteridophytes in reproductive health of tribal women of pachmarhi biosphere reserve, madhya pradesh, india. *International Journal of Pharmaceutical Sciences and Research*.

[4] Mangambu M., Kamabu V., Bola M. (2008). Les Plantes Médicinales Utilisées Dans Le Traitement De L’Asthme À Kisangani Et Ses Environs (Province Orientale, R.D.Congo). *Annales des Sciences*.

[5] Kolling M., Winkley K., von Deden M. (2010). “For someone who's rich, it's not a problem”. Insights from Tanzania on diabetes health-seeking and medical pluralism among Dar es Salaam's urban poor. *Globalization and Health*.

[6] Dougnon T. V., Deguenon E., Fah L. (2017). Traditional treatment of human and animal salmonelloses in Southern Benin: Knowledge of farmers and traditherapists. *Veterinary World*.

[7] Dougnon V., Legba B., Yadouléton A. (2018). Utilisation des plantes du Sud-Bénin dans le traitement de la fièvre typhoïde: rôle des herboristes. *Ethnopharmacologia*.

[8] Lègba B., Dougnon V., Ahoyo A. (2017). Exploration of the antibacterial and chemical potential of some Beninese pharmacopoiea traditional plants. *Microbiologia Medica*.

[9] Choi S. H., Kim J. Y., Mi Choi E. (2018). Heavy metal determination by inductively coupled plasma – mass spectrometry (ICP-MS) and direct mercury analysis (DMA) and arsenic mapping by femtosecond (fs) – laser ablation (LA) ICP-MS in cereals. *Analytical Letters*.

[10] Sarma H., Deka S., Deka H., Saikia R. R. (2011). Accumulation of heavy metals in selected medicinal plants. *Reviews of Environmental Contamination and Toxicology*.

[11] Tang R., Tian R., Cai J., Wu J., Shen X., Hu Y. (2016). Acute and sub-chronic toxicity of *Cajanus cajan* leaf extracts. *Pharmaceutical Biology*.

[12] Kévin Z. Z. A., Francis Y. A., Timothée O. A., Kévin Z. Z. A., Timothée O. A. (2018). Phytochemical and acute toxicity study of Cajanus cajan fabaceae. *European Journal of Biotechnology and Bioscience*.

[13] Zakaria Y., Azlan N. Z., Nik N. F., Muhammad H. Phytochemicals and acute oral toxicity studies of the aqueous extract of Vernonia amygdalina from state of Malaysia.

[14] Ekpo A. E., Eseyin O. A., Ikpeme A. O., Edoho E. J. (2007). Studies on some biochemical effects of vernonia amygdalina in rats. *Asian Journal of Biochemistry*.

[15] Etuk E. E., Francis U. F. (2003). Acute toxicity and efficacy of psidium guajava leaves water extract on salmonella typhi infected wistar rats. *Pakistan Journal of Biological Sciences*.

[16] Shreenidhi Ranjini P., Jeyamanikandan V. (2015). Evaluation of antibacterial, antioxidant and lipid degradation potential of Psidium guajava leaf extracts. *Journal of Pharmaceutical and Biomedical Sciences*.

[17] Olumese F. E., Onoagbe I. O., Eze G. I., Omoruyi F. O. (2016). Safety assessment of Uvaria chamae root extract: acute and subchronic toxicity studies. *Journal of African Association of Physiological Sciences*.

[18] Kroll M. T. (1999). Textbook of clinical chemistry. *Clinical Chemistry*.

[19] Ramaiah S. K. (2007). A toxicologist guide to the diagnostic interpretation of hepatic biochemical parameters. *Food and Chemical Toxicology*.

[20] Pramyothin P., Ngamtin C., Poungshompoo S., Chaichantipyuth C. (2007). Hepatoprotective activity of Phyllanthus amarus Schum. et. Thonn. extract in ethanol treated rats: in vitro and in vivo studies. *Journal of Ethnopharmacology*.

[21] Lawson-Evi P., Eklu-Gadegbeku K., Agbonon A. (2008). Toxicological assessment on extracts of Phyllanthus amarus Schum and Thonn. *Scientific Research and Essays*.

[22] Pour B. M., Latha L. Y., Sasidharan S. (2011). Cytotoxicity and oral acute toxicity studies of *lantana camara* leaf extract. *Molecules*.

[23] Fourasté I. (2013). Plantes Toxiques Sauvages Et Horticoles. *Institut Klorane*.

[24] Kalonda D. M., Tshikongo A. K., Koto F. K. (2016). Profil des métaux lourds contenus dans les plantes vivrières consommées couramment dans quelques zones minières de la province du Katanga. *Journal of Applied Biosciences*.

[25] Khankhane P. J., Jay G., Naidu V. S. G. R. (2012). Presence of heavy metals in medicinal weed species grown at contaminated sites. *Indian Journal of Weed Science*.

[26] Etornam Adukpo G., Kwaku Adotey J. P., Gyingiri Achel D. (2012). Trace and heavy metals analysis of Phyllanthus amarus and Phyllanthus fraternus in Ghana. *International Journal of Biological and Chemical Sciences*.

[27] Qishlaqi A., Moore F. (2013). *Statistical Analysis of Accumulation and Sources of Heavy Metals Occurrence in Agricultural Soils of Khoshk River Banks*.

[28] Gregor P., Newell A. (2004). Introduction. *Neuropsychological Rehabilitation*.

[29] Islam E. u., Yang X., He Z., Mahmood Q. (2007). Assessing potential dietary toxicity of heavy metals in selected vegetables and food crops. *Journal of Zhejiang University SCIENCE B*.

[30] Steenkamp V., Stewart M. J., Curowska E., Zuckerman M. (2002). A severe case of multiple metal poisoning in a child treated with a traditional medicine. *Forensic Science International*.

[31] Stewart M. J., Moar J. J., Steenkamp P., Kokot M. (1999). Findings in fatal cases of poisoning attributed to traditional remedies in South Africa. *Forensic Science International*.

[32] Steenkamp V., Von Arb M., Stewart M. J. (2000). Metal concentrations in plants and urine from patients treated with traditional remedies. *Forensic Science International*.

[33] Street R. (2012). Heavy metals in medicinal plant products — An African perspective. *South African Journal of Botany*.

[34] Rai V., Vajpayee P., Singh S. N., Mehrotra S. (2004). Effect of chromium accumulation on photosynthetic pigments, oxidative stress defense system, nitrate reduction, proline level and eugenol content of *Ocimum tenuiflorum* L. *Journal of Plant Sciences*.

[35] Ernst E. (2002). Toxic heavy metals and undeclared drugs in Asian herbal medicines. *Trends in Pharmacological Sciences*.

